# Multi-Scale Ecological Restoration Strategies to Enhance Water Conservation in Ruoergai on the Qinghai–Tibet Plateau

**DOI:** 10.3390/plants14071085

**Published:** 2025-04-01

**Authors:** Shiliang Liu, Yuhong Dong, Yongxiu Sun, Qingbo Wang

**Affiliations:** 1 State Key Laboratory of Regional Environment and Sustainability, School of Environment, Beijing Normal University, Beijing 100875, China; wangqingbo_27@foxmail.com; 2 Research Institute of Forestry, Chinese Academy of Forestry, Beijing 100091, China; yhongdong@163.com; 3 School of Petroleum and Environmental Engineering, Yan’an University, Yan’an 716000, China

**Keywords:** ecological restoration, multi-scale restoration, Yellow River Basin, driving mechanism, InVEST model

## Abstract

The Ruoergai Wetland is the highest and largest plateau peat swamp wetland in the world, providing more than 30% of the water for the upper reaches of the Yellow River. It performs vital regulatory functions in maintaining the quality and stability of the regional ecosystem of the Yellow River Basin. It is of great significance to study the spatial and temporal variability of water conservation services as well as ecological restoration and enhancement strategies at multiple scales. Based on field research, using the InVEST model, this study quantitatively assessed water conservation for a long period at the Ruoergai Wetland, proposing a strategy to improve water conservation capacity. The results showed that both grassland (mainly alpine meadow with Kobresia Willd and Cyperus papyrus) and wetland in the study area exhibited degradation. The proportions of significantly decreased, moderately decreased, slightly decreased areas were 50.64%, 16.81%, 11.64%, respectively. There were also significant changes in water conservation capacity from 2020 to 2023, with strong spatial heterogeneity. Average water conservation per unit area ranged from 52.70 to 211.99 mm/m^2^, with a decreasing trend. However, in the past 10 years, the area of soil erosion decreased by about 4735 km^2^. Although the soil erosion situation has improved to a large extent, there is still increasing soil erosion in some areas. Based on the field investigation, the intrinsic mechanisms of water conservation in alpine wetlands were elaborated, the driving forces behind the changes in water conservation functions were described, and further ecological restoration strategies were proposed from the perspectives of engineering measures, spatial zoning, and industrial structure.

## 1. Introduction

Water conservation constitutes a complex process of redistributing precipitation among vegetation, litter, and soil layers and is an important part of the water cycle system [1,2]. Climate change and human activities, as the main factors affecting the water resource system, also influence the characteristics of water conservation functions, and numerous studies have been carried out to quantitatively assess the impacts of climate change and human activities on runoff, water resource quantity, and hydrological processes in the watershed [3]. Water conservation functions refer to the ability of ecosystems to participate in the watershed water cycle, regulate hydrological processes, and generate ecological benefits in the process of water conservation, which is essential for maintaining ecosystem stability, while providing numerous regulating and provisioning services [4]. As an important ecosystem service, water conservation reflects the process and ability of ecosystems to maintain water reserves at a certain time and spatial scale, which has an important impact on regional ecosystem productivity, nutrient cycling, environmental purification, and other functions [5,6]. Due to the integrated interaction of ecosystems and hydrological processes at multi-temporal and spatial scales, water conservation involves a large number of components and eco-hydrological processes and has a variety of manifestations. In terms of its manifestations, water conservation functions primarily comprise water supply, runoff regulation, flood storage, water purification, soil and water retention, and local temperature regulation [7].

Based on the spatial variability of water conservation, spatial and temporal changes in water conservation in the upper part of the basin have a direct impact on the development of the regional ecosystem, which is of great significance for the prevention of flood and drought disasters and the rational development and utilization of water resources, as well as having an important impact on the production and life of human beings in the lower part of the basin [8,9]. From the perspective of ecosystem service functions, research in China on water conservation mainly focuses on changes in the amount or value of regional water conservation functions and their driving factors [6]. Although many valuable results have been achieved to analyze the temporal and spatial changes in water source containment at a large scale, research on how to enhance water conservation capacity is still relatively lacking [10].

The main methods for assessing water conservation include the forest canopy retention residual method, precipitation storage volume method, water balance method, and integrated water storage capacity method [1,11]. Liu et al. (2021) used the SWAT model to quantitatively assess the water conservation functions of different ecotypes in the middle and upper reaches of the Hun River [8]. Nie et al. (2010) regarded regional water storage and the amount of water supplied to river runoff as a reflection of regional water conservation capacity, which was quantified by the difference between rainfall and remote sensing evapotranspiration, and investigated the changes in water conservation amounts in the Qinghai–Tibet Plateau from 1982 to 2003, which revealed that the decrease in precipitation and the continuous enhancement of land surface evapotranspiration were the reasons for the weakening of water conservation capacity in the headwaters of Yarlung Zangbo River, and the study found that the water balance approach was the most appropriate way to assess water conservation [5]. The InVEST model has been widely used in the assessment of water conservation capacity in different regions [12]. This model has the advantages of lower data requirements and faster operating speeds, which is more suitable for the assessment of water conservation functions at the large basin scale. Moreover, the model can be further coupled with relevant land use change models to achieve the estimation of water conservation in historical and future periods, reflecting the impact of land use change on the water conservation capacity of ecosystems, and providing a scientific basis for regional water resource management and decision-making [13].

The Qinghai–Tibet Plateau is the birthplace of major rivers in China, such as the Yangtze, Yellow, Yarlung Tsangpo, and Lancang rivers, and in particular, the plateau peat swamp wetlands of the plateau have high water conservation capacity [14]. The plateau peat bog wetland is an intact depression basin formed by strong subsidence during the overall uplift of the Qinghai–Tibet Plateau at the end of the Tertiary and during the Quaternary [15]. The wetland is mainly composed of alluvial floodplains and river and lake sediments due to the soil-forming parent material, which is clay-heavy and difficult for soil moisture to infiltrate, with lush vegetation growth and lower residues that are not easy to decompose, whose organic matter accumulates as peat under smoky conditions, forming a peat layer of about 50 cm [16,17]. In the context of climate change, it is important to study the changes in the water conservation functions of highland peat bog wetland watersheds in order to safeguard the water security of the Qinghai–Tibet Plateau and its downstream watersheds. Specifically, the Ruoergai Wetland is the highest and largest plateau peat bog wetland in the world and is the most important water conservation area of the Yellow River. This region is also an important part of the Qinghai–Tibet Plateau ecological barrier. The valleys in the Ruoergai Basin have the most primitive and representative peat bog wetland in the Qinghai–Tibet Plateau [18], which is known as the “Yellow River Reservoir”. It constitutes a vital ecological security-sensitive region within the Yellow River Basin and even the nation, and the examination of variations in the water conservation capacity of this area possesses significant ecological value [19].

Water conservation is highly sensitive to human activities [20]. To protect its regional ecological function, systematic, comprehensive, and scientific ecological restoration measures are necessary. These measures should include clarifying ecological function zones, establishing clear protection directions, optimizing spatial layouts, implementing classified guidance, and coordinating regional adaptive management. This will effectively enhance the protection of the Ruoergai Wetland’s water conservation and runoff regulation functions [21]. However, there is still a lack of research in this area on how to enhance water conservation capacity based on the pathway of ecological restoration [22]. For the Ruoergai Wetland, due to the influence of complex topography and geomorphology, drastic climate change, and frequent natural disasters, the trend of declining functions of important ecological barriers, such as wetland shrinkage, grassland degradation, soil erosion, riverbank erosion, and forest reduction, in the localized areas of Ruoergai has not yet been fundamentally changed [23,24]. Xiong et al. (2011) analyzed changes in the water conservation functions of the Ruoergai Wetland and its causes by using soil water content as a typical index [7]. Based on the improved LPJ dynamic vegetation model, Yin et al. (2016) investigated the impact of climate change on water conservation in the Yellow River source area from 1981 to 2010, but the study from the perspective of ecosystem services is still insufficient [25].

This study quantitatively assessed the spatial and temporal variability of the water conservation in the Sichuan section of the upper reaches of the Yellow River. Based on the assessment of water conservation in the Sichuan section of the upper reaches of the Yellow River, we analyzed the trends in wetland degradation, grassland degradation, and soil erosion, and elucidated the driving mechanisms behind the loss of water conservation in the Ruoergai wetland. At the same time, based on the field investigation, multi-scale ecological restoration strategies are proposed, with a view to providing a scientific basis for the ecological protection and engineering layout of the Ruoergai Wetland and improving water conservation capacity.

## 2. Results

### 2.1. Degradation of Grasslands and Wetlands

Due to climatic, geological, geomorphological, and biological constraints, land use in the district is dominated by meadows, with a share of about 57.96%, followed by grasslands, with a share of 33.46%. Herbaceous wetlands and deciduous broad-leaved shrub forests accounted for 5.99% and 1.17%, respectively. The area of other types of land use only accounted for 1.41% of the total land area. Meadows are mainly found in Changsha Gongma Township in Shiqu County, Kujima Township in Aba County, and Jaiman Township in Ruoergai County.

Based on the field survey, since the 1960s, Ruoergai grassland has been degraded as a whole, and due to its good conditions for grassland animal husbandry, grazing is the main source of the economy. However, in recent decades, the problem of grazing overload has become more and more serious, with the overload rate reaching 78.3%, and the degradation of the grassland accelerated. Overgrazing has accelerated the degradation of grassland. Although a large number of ecological projects have been devoted to grassland restoration, vegetation restoration is still facing serious challenges due to the aggravation of grass–animal conflicts. The main manifestations are the decline in grass production per unit area, the lowered proportion of high-quality pasture, and the substantial reduction in grassland vegetation cover and pasture plant species. Overgrazing in some areas led to the degradation of large areas of natural vegetation and pasture, and the degradation of grasslands has caused herders to shift from sedentary to nomadic herding, or even to rent grasslands for grazing. On the other hand, the degradation of pastureland has reduced the resources of grazable grassland, and herders have had to increase pressure on pastureland in order to maintain their livelihoods, leading to further degradation of the grassland and the intensification of disasters. In the Ruoergai region, 26% of the area is still showing a decreasing trend in vegetation cover, such as in Jiangmou Township, Amu Township, and Longri Township, and 24% of the area is not showing significant recovery of vegetation, such as in Banyou Township and Anqu Township. In 2020, by watershed, the share of degraded grassland in the Heihe, Baihe, and Jagchu river basins will reach 18.52%, 21.10%, and 70.38%, respectively.

**Figure 1:**
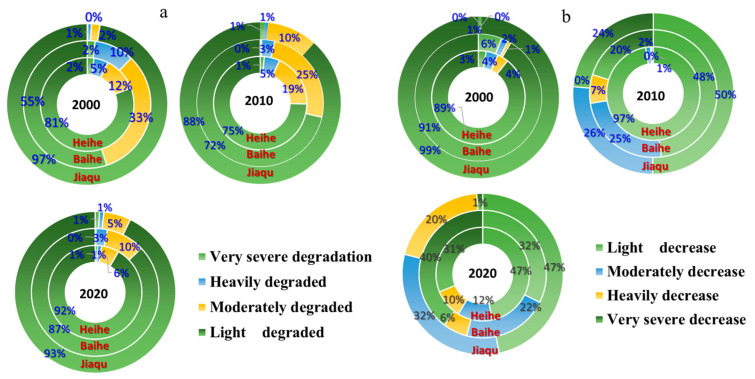
Levels and proportions of grassland (**a**) and wetland (**b**) degradation in different watersheds (Heihe River, Baihe River, and Jiaqu River) in Ruoergai.

From 2000 to 2020, the average water yield per unit area of the Ruoergai Plateau ranged from 52.70 to 211.99 mm/m^2^, and the proportion of the area that remained in the range of 100–200 mm/m^2^ was 63.66%, while the proportion of the area with a water yield of >200 mm/m^2^ was very small. The proportions of areas with significantly decreased, moderately decreased, slightly decreased, almost unchanged, slightly increased, moderately increased, and significantly increased average water conservation per unit area were 50.64%, 16.81%, 11.64%, 5.20%, 4.52%, 7.31%, and 3.88%, respectively. The water conservation functions of Longri Township, Anqu Township, Charlie Township, and Amu Township in the project area have been improved (2585–7646 t). The water conservation functions of Changsha Gongma Township, Gluttony Township, Axi Township, Zazhi Township, and Maixi Township are still low (Figure 1).

**Figure 2:**
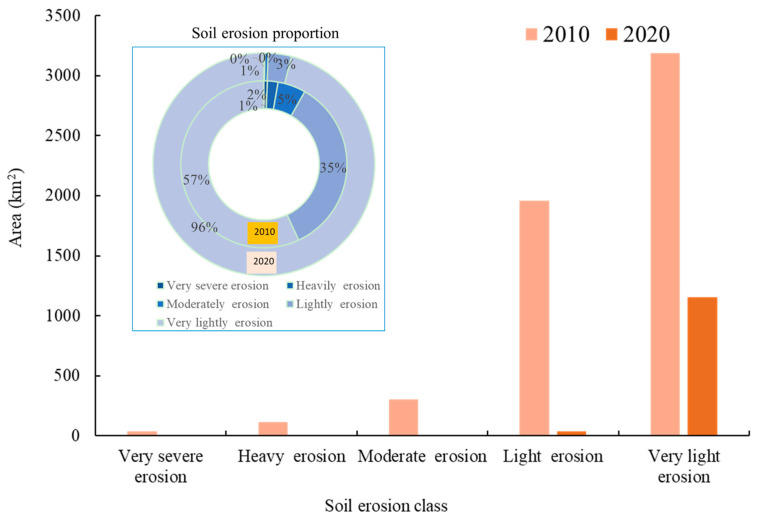
Differences in soil erosion classes in the study area based on survey data.

Based on the soil erosion survey data of the counties in the study area, the current soil erosion situation in 2010 was further compared, and the results show that the area of soil erosion decreased at a rate of 676.43 km^2^ per year, and in the past 10 years, the area of soil erosion decreased by about 4735 km^2^, with the regional erosion situation being curbed to a greater extent. But at present, the area of soil erosion is controlled in a certain way, and at the same time, the phenomenon of increasing soil erosion levels is still present in part of the area. The key species for soil erosion control are *Kobresia pygmaea*, *Stipa bungeana*, and *Carex praeclara*. However, there is still a certain amount of soil erosion, and there are still some areas where the soil erosion level is aggravated (Figure 2). Although the grassland desertification has been treated, the speed of recovery is still smaller than the speed of sanding, so the deterioration trend has not been improved on the whole, and the water conservation capacity has not been effectively repaired. In the study area, the amount of soil preserved in the towns of Tounenwa and Truong Sa Gonma is still low, while the amount of soil preserved in the towns of Amu and Maikun is high.

### 2.2. Trends in Water Conservation in the Ruoerge Region

**Figure 3:**
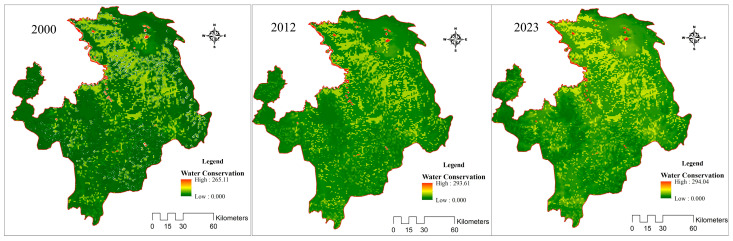
Spatial and temporal variation of water conservation in Ruoergai region.

Statistical data show that the average water conservation in the Ruoergai Wetland was 230.1 mm (3.90 billion m^3^) from 1971 to the present, with the highest amount being 348.1 mm (5.91 billion m^3^) in 1975, and the lowest being 121.4 mm (2.06 billion m^3^) in 2002. Based on the spatial data, this study calculated the water conservation index of the Ruoergai Wetland from 2000 to 2023, as shown in Figure 3. The results show that the distribution of water conservation in the study area has obvious spatial heterogeneity, and the amount of water conservation in general shows a trend of being high in the south and low in the north, and decreasing from the southwest to the northeast; the ecosystems of shrub forests, coniferous forests, and grasslands in areas of high precipitation in the southwest region play a very important role in the conservation of water. The water conservation values were 8.96, 15.02, and 19.8 mm for 2000, 2012, and 2023, respectively. Areas with high water conservation are mainly concentrated in the western and southern parts of Aba and Hongyuan counties; areas with medium water conservation are mainly located in the northern and northeastern parts of Aba and Hongyuan counties and in the central part of Ruoergai county; Songpan and Shiqu counties, as well as the eastern and western parts of Ruoergai county, have low water conservation.

**Figure 4:**
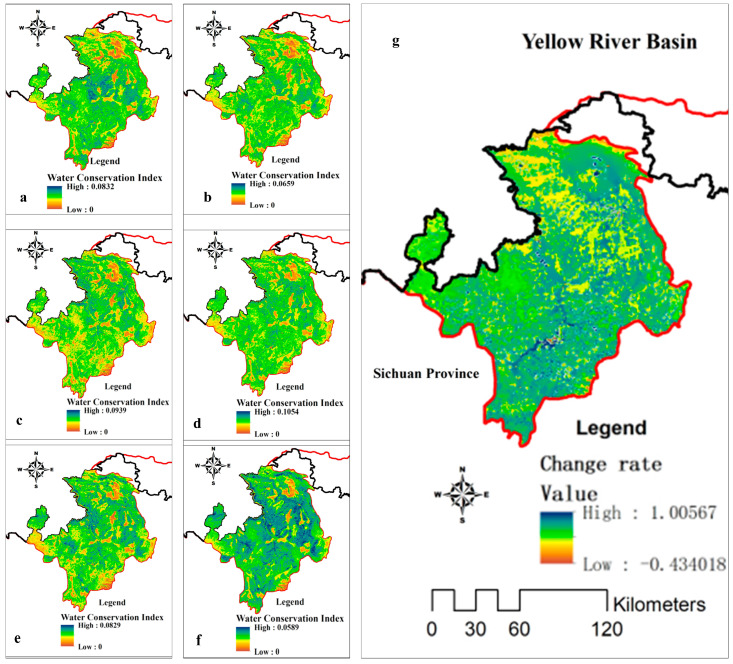
Spatial and temporal variability of water conservation function index. (**a**–**f**) indicate the indexes of 2000, 2005, 2010, 2015, 2020, and 2023; (**g**) is the change rate of the function index.

Based on the trend analysis, the spatial changes in the water conservation function index in the Ruoergai region were obtained, and it can be seen that, on the whole, the water conservation function index in the south tends to increase in general, and the type of grassland around the subalpine forests especially has significantly improved (*p* < 0.05) (Figure 4). The subalpine coniferous forest is dominated by species like *Abies faxoniana*, *Picea purpurea*, and *Picea asperata*, with understory plants such as *Fargesia nitida*. The change rate ranges from −0.43 to 1.00. For alpine meadows, in the context of the ecological protection and restoration of wetland functional areas, the wetland water conservation function index has been gradually and effectively protected, and the water conservation functions of Longzhi Township, Anqu Township, Chari Township and Amu Township have been improved. The water conservation function in Changsha Gongma Township, Gluttony Township, Axi Township, Zazhi Township, and Maixi Township is still low; these are areas that needs urgent wetland management at the present time.

### 2.3. Multi-Scale Ecological Restoration Strategies

**Figure 5:**
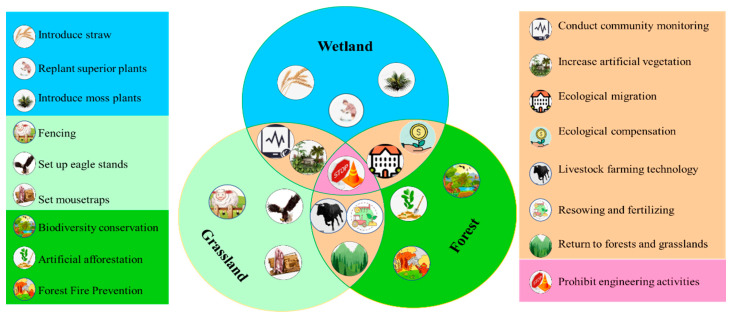
Multi-scale ecological restoration model of Ruoergai Wetland (Drawn by authors).

Based on the results of analyzing the spatial differentiation and change trends in water source containment, this study further combines with the field research. This study proposes ecological restoration strategies from the perspectives of engineering measures, spatial zoning, and industrial structure. From the species, population, ecosystem, and landscape levels, measures should be adopted, such as the selection of excellent pasture species, community configuration of different wetland types, forest and grass restoration and moisture maintenance, landscape pattern optimization, and geomorphological reshaping. Wetland restoration projects should be carried out, such as returning pasture to grass, rotational grazing and grazing bans, raising the groundwater level, and desertification control. The implementation of Nature-based Solutions (NbS) in ecological protection and restoration should be promoted to enhance wetland connectivity, optimize landscape spatial patterns, maintain the safety pattern of biodiversity conservation, and enhance landscape sustainability. Figure 5 illustrates conservation strategies for wetland, grassland, and forest ecosystems with different measures. The overlapping areas highlight shared measures such as conducting community monitoring, ecological migration, and introducing moss plants, emphasizing integrated conservation efforts across ecosystems.

**Figure 6:**
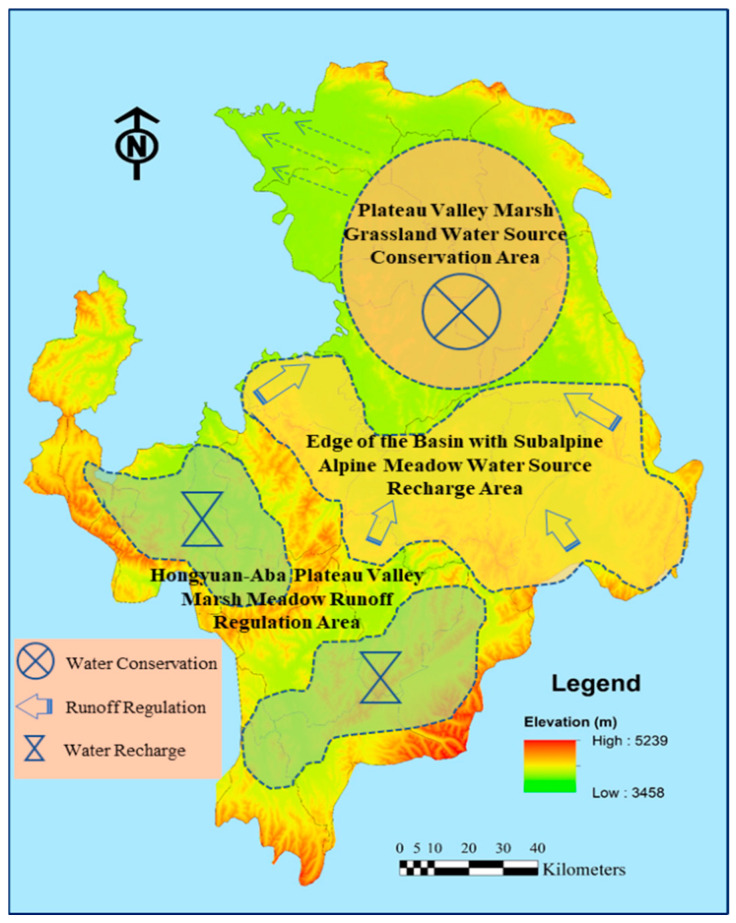
Water conservation promotion zoning of Ruoergai Wetland (drawn by authors).

From the perspective of spatial zoning, ecological space zones are scientifically divided, the ecological functions and protection directions of each zone are clarified, the spatial layout is optimized, and classified guidance is implemented. According to the characteristics of different areas, such as the swampy meadows in the broad valleys of hills and plains, the grasslands in the broad valleys of hills and plains, the swampy meadows in the valleys between the hills and plains of plateaus, and the alpine and subalpine meadows, etc., targeted protection and restoration measures are carried out to improve the stability of the ecosystem and enhance the functions of water conservation and runoff regulation, and the results of the functional partition are shown in Figure 6.

According to our field investigation, from the perspective of industrial structure development and according to the law of wetland protection and socio-economic coupling and development, strategies for eco-industry development in different human activity zones will be clarified. For areas suitable for agriculture and animal husbandry development, it is better to optimize the grazing layout according to the grassland’s carrying capacity and build high-quality and high-yield artificial forage bases and forage seed breeding bases. For areas suitable for tourism development, it is necessary to promote the transformation and upgrading of the local economy relying on the National Wetland Park. For the plateau specialty industry area, the development of “photovoltaic + ecological environmental governance” in energy use should be promoted, which is the integration of photovoltaic energy systems with environmental restoration and management practices. This approach can modify local microclimates, improve soil moisture, and support vegetation growth, thereby contributing to the restoration of degraded ecosystems. In terms of agricultural and animal husbandry development, we must build modern agricultural and animal husbandry industrial parks and promote the high-quality development of the ecological agriculture and animal husbandry industries.

## 3. Discussion

### 3.1. Mechanisms of Water Conservation in Alpine Wetlands

**Figure 7:**
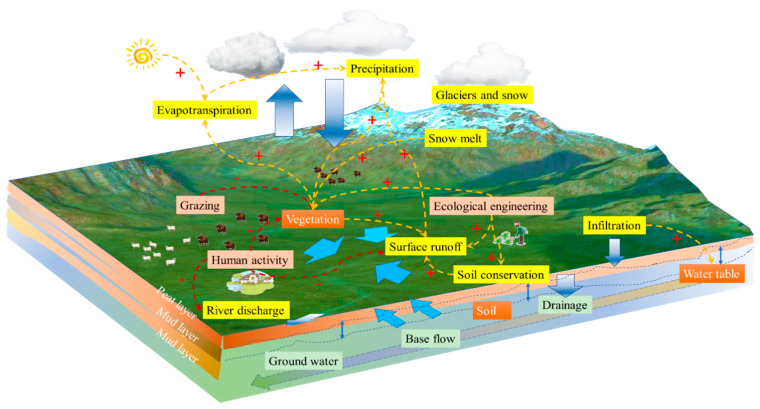
Mechanisms of change in Ruoergai’s water conservation (+: positive effect, −: negative effect) (drawn by authors).

Water conservation is an important mechanism for maintaining the water cycle and ecological balance. It involves a variety of natural processes and human activities that work together to store, cycle, and utilize water. In this study, the mechanisms of change in water conservation are plotted in Figure 7, from which it can be seen that the mechanisms of water conservation include elements such as precipitation, glacier and snow accumulation, evapotranspiration and transpiration, snowmelt, grazing, ecological engineering, vegetation cover, infiltration, surface runoff, human activities, soil conservation, groundwater level, river discharge, drainage, soils, base flow, and groundwater. Precipitation is the starting point for water conservation, as it provides initial moisture to the surface and subsurface [26]. Glaciers and snow act as natural reservoirs that store large amounts of freshwater resources. Evaporation and transpiration are part of the water cycle, and they release water from the surface and plants into the atmosphere. Snowmelt recharges rivers and groundwater, especially in spring and summer. Human activities such as grazing may affect vegetation cover and soil structure, which in turn affects water conservation capacity [27].

Ecological engineering and vegetative cover contribute to water retention and groundwater recharge by increasing soil permeability and reducing surface runoff. Soil conservation and maintenance of the water table are key to water conservation, as they reduce water loss and increase water availability. River discharge and drainage systems play an important role in regulating water allocation and preventing flooding. Base flows and groundwater are a stable supply of water resources and are essential for maintaining ecosystems and human societies. Therefore, water management is a complex process that requires integrated consideration of the impacts of natural conditions and human activities to achieve sustainable management and use of water resources [28].

The main problems facing water conservation in the Ruoergai include grassland degradation, wetland shrinkage, soil erosion, permafrost thawing, and other major ecological and environmental problems. It has been shown that the wetland shrinkage of Ruoergai peat swamps became obvious in the 1960s, and intensified after the 1980s, with the transition or change from perennial waterlogged swamps to seasonal waterlogged swamps, the lowering of the surface water level of peat swamps, the reduction in vegetation cover and weakening of its permeability, and the decrease of grass production by about 20% compared with that of the 1960s [29]. Wetland degradation and shrinkage mainly occurred in the Ruoergai Wetland and the Baihe and Heihe river basins, and wetland degradation was mainly transformed into grassland [30]. The degradation of the Ruoergai Wetland on the Qinghai–Tibet Plateau has led to significant consequences, such as decreasing water supply and flood control ability in the Yellow River, reducing biodiversity level, and diminishing habitat area. Additionally, the degradation has decreased the ecosystem’s ability to store carbon, exacerbating climate change effects. Lastly, the decline in ecosystem services has negatively impacted local livelihoods, particularly those dependent on livestock farming and other natural resources.

### 3.2. Temporal and Spatial Evolution of Human Impacts on Water Conservation in the Ruoergai Wetlands

The Ruoergai Wetlands are increasingly at risk from climate change and anthropogenic disturbances, with a decrease in precipitation, an increase in potential evapotranspiration, and a trend toward a colder and drier climate; there has been a trend of increasing mean temperature in marshy wetlands, with an overall rate of change of 0.28 °C per decade, which is significantly higher than the global average rate of increase in temperature, leading to a decrease in the amount of water contained and the rate of water containment. It has also been shown that runoff from the Ruoergai Wetland basin will continue to show a decreasing trend under different climate change scenarios until 2050 [31].

The continuing increase in demand for grassland resources caused by population growth is a long-term dilemma facing the Ruoergai Wetlands, where the population has more than doubled in the space of 60 years. Man-made activities have intensified, livestock development is still in rough development mode, and the increased demand for grazing will open and drain the swamps, leading to the disappearance of wetlands. In addition, the uncontrolled development of peat resources, overgrazing, and tourism activities have led to the destruction of wetland vegetation, loss of biodiversity, and disruption of the virtuous cycle of the wetland ecosystem, leading to a reduction in its water conservation functions. In addition, the increase of rodent infestation is also an important factor in grassland degradation, especially after the artificial thinning of the wetland; the area of rodent infestation in the Ruoergai plateau has obviously expanded, and rodent infestation has spread from the distribution area of meadow soils to the original distribution area of swampy soils and peat soils [32].

Ecological protection and restoration, in the context of Ruoergai Wetland conservation, have gradually ensured effective protection for the wetland. Many measures have been implemented to improve soil and water conservation, with notable effectiveness, such as blocking drainage canals, recovering vegetation, and planting pioneering species. For species, key species such as *Elymus dahuricus* and *Medicago sativa*, *Hippophae rhamnoides*, and *Kobresia* spp. play important roles in soil erosion control and enhancing water conservation capacity. These species fix the soil through their root networks, thereby reducing soil and water loss. These measures are evident in improved soil retention and water conservation capacities, which have shown an increasing trend from 2010 to 2020. At present, vegetation recovery in the Ruoergai Wetland area has reached 6400 ha, and the implementation of the “Integrated Protection and Restoration Project of Mountain, Water, Forest, Field, Lake, Grass, Sand and Ice” in the Upper Yellow River Ruoergai Grassland Wetland in Sichuan in 2022 will promote the ecological protection and restoration of the region through systematic protection and management, from the source, in a holistic, systematic, and integrated way.

### 3.3. Ecological–Economic–Social Dimensions of Water Conservation Capacity Enhancement

**Figure 8:**
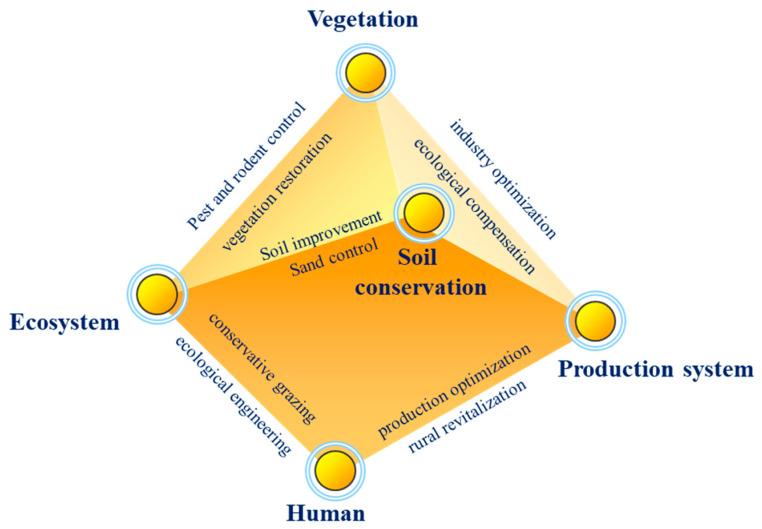
Dimensions for the enhancement of water conservation capacity (drawn by authors).

Wetland water conservation enhancement relates to different dimensions of ecology–economy–society. Figure 8 shows the different factors influencing water conservation, ecosystems, vegetation, animal husbandry, and human activities, which are all closely related to wetland water conservation capacity. Only through scientific management and the rational use of wetland resources can the economic development and social progress of rural areas be promoted, and the harmonious coexistence of human and nature can be achieved [33].

In order to strengthen the ecological protection and restoration of the Ruoergai Wetland, a series of strategies need to be implemented to achieve the coordinated development of ecology, economy, and society. Combined with the research of the ecological restoration project, measures to enhance water conservation capacity from multiple perspectives and scales are proposed. These include the scientific planning of wetland zoning, clarifying the ecological function and protection direction of each zone, optimizing the spatial layout, combining wetland protection with socio-economic development, formulating eco-industry development strategies, and carrying out wetland protection and restoration based on the integrated protection and restoration project of mountains, water, forests, fields, lakes, grasses, sand, and ice [34].

Since the Ruoergai Wetland belongs to different provinces, it is necessary to set up a cross-regional management organization to unify and coordinate wetland protection and restoration work. In the process of ecological restoration, it is necessary to further promote nature-based solutions (NbS) to enhance wetland connectivity and landscape sustainability, and at the same time, it is necessary to broaden the channels of multi-dimensional integrated governance to enhance the vitality of the governance system. Social capital and local residents are encouraged to participate in ecological protection and restoration, and social capital is guided to focus on key engineering restoration areas [35].

In addition, it is necessary to further strengthen awareness of the protection of farmers, herdsmen, and tourists in the ecological restoration of wetlands, and improve conscientiousness regarding protection and restoration. Local governments should improve the financial input guarantee mechanism, perfect the ecological compensation mechanism, and encourage social organizations and individuals to participate in ecological protection and restoration or, later, operation and maintenance. Also, local governments should adopt one-time compensation for the herdsmen in the core functional area, compensation for seasonal grazing restrictions and wetland return, compensation for forbidden grazing and wetland return, and compensation for the balance between grass and livestock, to carry out the wetland ecological protection effectively [36]. Further, a three-pronged farming method of “grazing + supplemental feeding + captive breeding” for yak was explored to reduce grassland trampling damage and pasture waste and to encourage herders to change their production methods and increase their income, so that an ecological environment that involves all people’s participation in building and sharing will gradually be formed [37]. In addition, it is suggested that herders in the core functional areas of the wetland should be relocated to the urban periphery to reduce their dependence on wetland pastures and accelerate the urbanization process. Through these ecological restoration measures, the objective is to achieve long-term sustainable development in the Ruoergai Wetland, protect biodiversity, enhance ecosystem service functions, and promote local economic and social development. Moreover, improved ecosystem health has had positive socioeconomic implications for local communities, with increased tourism and opportunities for sustainable livelihoods. However, challenges remain, including the need for continuous monitoring and adaptive management to address emerging issues such as invasive species and changing climate conditions.

## 4. Materials and Methods

### 4.1. Study Area

**Figure 9:**
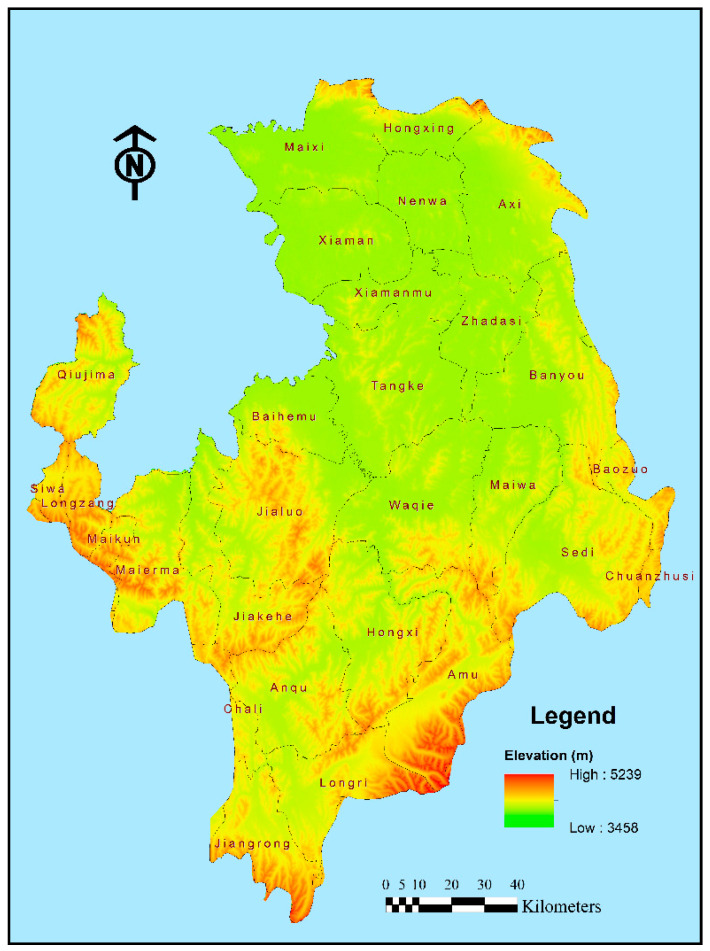
The DEM map of study area.

The Ruoergai Wetland (32° N–35° N, 98° E–105° E) is located at the eastern edge of the Qinghai–Tibet Plateau, where the main stream of the Yellow River is 174 km long, accounting for 3% of the total length of the whole Yellow River, and the watershed area is 18,700 km^2^, accounting for 2% of the total area of the Yellow River Basin (Figure 9). The study area has a multi-year average water volume of 4.76 billion m^3^ (surface runoff), total annual precipitation of 12.15 billion m^3^, and total multi-year average water resources of 4.89 billion m^3^, accounting for 8% of the Yellow River Basin, which is an important part of the “Chinese Water Pagoda”. The region is strategically important for the ecological security of the Yellow River Basin as a whole. The average altitude is about 3500 m. The main types of geomorphologies are medium-high mountains, hilly plateaus, terraces, river floodplains, wide valleys, and lake depressions. The vegetation in the study area belongs to the alpine vegetation zone of the Qinghai–Tibet Plateau, and the vegetation types mainly include swamp vegetation, meadow vegetation, scrub vegetation, cold desert vegetation, and a few sporadic areas of forest vegetation [38]. The vegetation belongs to the alpine vegetation zone, with the main types including swamp vegetation dominated by *Kobresia littledalei* and *Kobresia tibetica*, meadow vegetation dominated by *Kobresia pygmaea* and *Polygonum sphaerostachyum*, and scrub vegetation with species like *Hippophae rhamnoides*. These dominant plant species play crucial roles in soil erosion control and water conservation. The study area belongs to the continental plateau cold–temperate humid and semi-humid monsoon climate, and compared with other major wetlands in the world, the Ruoergai Wetland has special climatic characteristics. It belongs to the continental plateau climate, with an average annual temperature of 0.9 °C, a maximum average temperature of 10.9 °C in July, a minimum average temperature of −10.3 °C in January, an average annual precipitation fluctuating between 657 and 730 mm, 80% of which is concentrated in the period from May to August, and an average relative humidity of about 65%. Perennial low temperature is the main climate characteristic of the Ruoergai Wetland. In this region, under the opportunity of “Western Development” and “The construction of the ecological barrier in the middle and upper reaches of the Yellow River” in 2000, ecological projects such as returning farmland to forests, natural forest protection, returning pasture to grassland, wetland protection, and soil erosion control have been implemented in Aba Prefecture, Sichuan Province and have achieved certain results. However, affected by climate change and human activities, the region still suffers from the degradation of wetland functions, soil erosion, and grassland degradation and sanding [32].

### 4.2. Data Sources and Methods

#### 4.2.1. Data Sources

The data required for the InVEST model are precipitation, evapotranspiration, land use data, and the geographic elevation model [39]. The specific data sources are shown in Table 1, respectively. All raster data were uniformly projected into the projected coordinate system Krasovsky_1940_Albers, cropped to the size of the study area by using the extract by mask tool in ArcGIS, and resampled to 30 m × 30 m.

#### 4.2.2. InVEST Model

Based on the calculation results from the water production module of the InVEST model, taking into account the influences of soil permeability and the topographic differences across different land use/cover types on surface runoff, the amount and depth of each unit’s water conservation were calculated using topographic indices and soil base data, which can better express the spatial distribution of water conservation in the watershed and the main factors affecting the amount of water conservation. According to the principle of water balance, the basic method of the InVEST model for finding water yield is to use the amount of rainfall per unit area minus the actual evapotranspiration, not taking into account the interaction between groundwater and surface water [40]. The InVEST water yield assessment is based on the assumptions of Budyko’s water–heat coupling equilibrium and annual rainfall data, and the formula for water yield is as follows:

(1) $$Y_{xj}=\left(1-\frac{{\mathrm{AET}}_{\mathrm{xj}}}{P_{x}}\times P_{x}\right)$$

where *Y*(*x*) denotes the annual water production of grid cell *x* in the study area; AET(x) denotes the actual inter-annual evapotranspiration of grid cell *x* in the study area; and P(x) denotes the annual rainfall of grid cell *x* in the study area.

In the equation,

(2) $$\frac{{\mathrm{AET}}_{xj}}{P_{x}}=\frac{1+\omega xP_{xj}}{1+\omega xP_{xj}+\frac{1}{R_{xj}}}$$

(3) $$\omega =Z\frac{{\mathrm{AWC}}_{x}}{P_{x}}$$

$${\mathrm{AWC}}_{x}=\min\left(\mathrm{maxsoil}\;\mathrm{depth},\;\mathrm{root}\;\mathrm{depth}\right)\times {\mathrm{PAWC}}_{x}$$

$${\mathrm{PAWC}}_{x}=54.409-0.132\times sand-0.003\times \left(\mathrm{sand}\right)\times 2-0.055\times silt-0.006\times {\left(silt\right)}^{2}\;-0.738\times clay+0.007\times {\left(clay\right)}^{2}-2.688\times \mathrm{OM}\;+0.501\times {\left(\mathrm{OM}\right)}^{2}\;$$

(4) $$R_{xj}=\frac{K_{xj}ET0_{x}}{P_{x}}$$

where: R*_xj_* is the ratio of potential evapotranspiration to precipitation; ωx is a non-physical parameter that expresses the relationship between climate and soil properties; AWCx is the effectively utilized water content of vegetation (mm); max soil_depth is the maximum soil depth (mm); root_depth is the root depth (mm); PAWCx is the amount of water available to the plant (%); sand is the soil; ET0x is the potential evapotranspiration of unit x (mm); and Kxj is the evapotranspiration coefficient of a vegetation type.

Based on the estimation of the water balance in the InVEST model, the amount of water contained in the study area was calculated by combining the surface flow coefficient, the topographic index, and the saturated hydraulic conductivity of the soil, and the calculation formula was as follows [12].

(5) $$Re=min\left(1,\frac{249}{velocity}\right)\times min\left(1,\frac{0.9\times TI}{3}\right)\times min\left(1,\frac{ks}{300}\right)\times Y_{xj}$$

(6) $$TI=1g(\frac{{\mathrm{Drainage}}_{a}\mathrm{rea}}{{\mathrm{soil}}_{d}\mathrm{epth}\times {\mathrm{percent}}_{s}\mathrm{lope})}$$

(7) $$ks=609.6\times 10^{\left[-0.6+0.012\times sand-0.006\times clay\right]}$$

In the formula, Re is the water source capacity (mm); velocity is the flow rate coefficient; TI is the topographic index; ks is the soil’s saturated hydraulic conductivity (mm/d); Yxj is the water yield (mm); Drainage_area is the cumulative number of grids in catchment catchment; soil_depth is the depth of the soil (mm); percent_slope is the percent slope (%); sand is soil sand content (%); clay is soil clay content (%).

The water conservation function index is the ratio of the amount of water per unit area to the amount of rainfall per unit area. The results of evaluations of water conservation function are usually classified into different grades according to area, with a water conservation function index.

#### 4.2.3. Trend Analysis

The trend in water conservation function was calculated using a one-dimensional linear regression method based on time series analysis [40]. We calculated the annual water conservation function from 2000 to 2023. Its trend line was fitted using the least squares method, and the reliability of the fitted equation was determined using the F-test. On this basis, the rate of change in water conservation capacity was calculated on a pixel-by-pixel basis.

(8) $$b=\frac{\sum_{i=1}^{n}x_{i}Y{\left(x\right)}_{gi}-\frac{1}{n}\left(\sum_{i=1}^{n}x_{i}\right)\left(\sum_{i=1}^{n}Y{\left(x\right)}_{gi}\right)}{\sum_{i=1}^{n}x_{i}^{2}-\frac{1}{n}{\left(\sum_{i=1}^{n}x_{i}\right)}^{2}}$$

(9) $$a=Y{\left(\bar{x}\right)}_{g}-b\times \frac{\sum_{i=1}^{n}x_{i}}{n}$$

(10) $$R=\frac{\left(x_{n}\times b+a\right)-\left(x_{1}\times b+a\right)}{x_{1}\times b+a}\times 100$$

where xi denotes the ith year in the study period; b is the slope of the trend line of change; a is the intercept; R is the rate of change of *Y*(*x*); and x1 and xn are the starting and ending years of the study period, respectively. The study period of this research is from 2000 to 2023.

#### 4.2.4. Field Survey and Ecological Restoration Project Research

This study carried out research work and data collection in the study area in August 2023, mainly to collect relevant data and information from the study area, such as information related to the water resources bulletin, agriculture, and animal husbandry. Typical townships were selected for the study of current grassland ecological degradation, grassland utilization, and animal husbandry development. In order to obtain first-hand data and information, we researched the relevant departments, such as the Animal Husbandry and Forestry Bureau, Meteorological Bureau, Development and Reform Bureau, and Land and Resources Bureau of Maqu County, Aba County, Hongyuan County, and Ruoergai County, centered on the county towns of Maqu, Aba, Hongyuan, and Ruoergai, and conducted interviews with the relevant staff. At the same time, combined with the ecological restoration project carried out in Ruoergai, we carried out research on the effectiveness of ecological restoration and ecological compensation.

## 5. Conclusions

This study utilizes model simulation and field research to analyze water conservation changes in the Ruoergai Wetland and further proposes multi-scale ecological restoration strategies through model simulation and field research. This study reveals the driving mechanisms behind the changes in water conservation capacity, emphasizing the need to consider both natural conditions and human activities. Specifically, the study identifies the degradation of grasslands and wetlands, the decrease in water conservation capacity, and the impact of soil erosion as key issues. Between 2020 and 2023, there were notable shifts in water conservation capacity, marked by significant spatial variability. The average water conserved per unit area exhibited a general decline. To address these, the research proposes multi-scale ecological restoration strategies at the species, ecosystem, and landscape levels. It is necessary to implement the “Integrated protection and restoration project of mountains, water, forests, fields, lakes, grasses, sand and ice” from the perspectives of engineering measures, spatial zoning, and industrial structure to optimize ecological spatial zoning. In addition, Nature-based Solutions (NbS) are promoted to enhance wetland connectivity and landscape sustainability. In conclusion, the protection and enhancement of the water conservation functions of the Ruoergai Wetland is a multi-dimensional, interdisciplinary, and complex process that requires the concerted efforts of the government, scientific research institutes, local communities, and all sectors of society. By effectively improving the water conservation capacity of the Ruoergai Wetland, the study aims to promote the sustainable development of the local economy and society, achieving a win-win situation between ecological protection and economic development.

## Figures and Tables

**Table 1:** Data sources and basic information.

Name	Data Sources	Resolution/m
Land use type	Data Centre for Resource and Environmental Sciences, Chinese Academy of Sciences (https://www.resdc.cn/)	30
Evaporation	National Earth System Science Data Centre (http://www.geodata.cn)	30
River data	Resource Environmental Science and Data Centre (https://www.resdc.cn)	——
Slope direction	Geospatial data cloud (http://www.gscloud.cn/)	30
Elevation	Geospatial data cloud (http://www.gscloud.cn/)	30
Soil data (soil depth, soil saturated hydraulic conductivity, soil sand content, soil clay content)	World Soil Database (HWSD) (Harmonised World Soil Database version 1.1, http://data.tpdc.ac.cn, accessed on 31 March 2025)	1000
Vegetation cover	Cloud platform for resource and environmental data (http://www.Resdc.cn/doi/doi.aspx?doiid=49, accessed on 31 March 2025)	30

## Data Availability

The original contributions presented in the study are included in the article. Further inquiries can be directed to the corresponding authors.
